# What factors influence the willingness and intensity of regular mobile physical activity?— A machine learning analysis based on a sample of 290 cities in China

**DOI:** 10.3389/fpubh.2025.1511129

**Published:** 2025-01-23

**Authors:** Hao Shen, Bo Shu, Jian Zhang, Yaoqian Liu, Ali Li

**Affiliations:** ^1^School of Architecture, Southwest Jiaotong University, Chengdu, China; ^2^School of Design, Southwest Jiaotong University, Chengdu, China; ^3^SWJTU-LEEDS Joint School, Southwest Jiaotong University, Chengdu, China; ^4^Information and Network Management Center, Xihua University, Chengdu, China

**Keywords:** physical activity, willingness and intensity, socioeconomic factors, geographical environmental factors, built environmental factors, machine learning, mechanisms of influence

## Abstract

**Introduction:**

This study, based on Volunteered Geographic Information (VGI) and multi-source data, aims to construct an interpretable macro-scale analytical framework to explore the factors influencing urban physical activities. Using 290 prefecture-level cities in China as samples, it investigates the impact of socioeconomic, geographical, and built environment factors on both overall physical activity levels and specific types of mobile physical activities.

**Methods:**

Machine learning methods were employed to analyze the data systematically. Socioeconomic, geographical, and built environment indicators were used as explanatory variables to examine their influence on activity willingness and activity intensity across different types of physical activities (e.g., running, walking, cycling). Interaction effects and non-linear patterns were also assessed.

**Results:**

The study identified three key findings: (1) A significant difference exists between the influencing factors of activity willingness and activity intensity. Socioeconomic factors primarily drive activity willingness, whereas geographical and built environment factors have a stronger influence on activity intensity. (2) The effects of influencing factors vary significantly by activity type. Low-threshold activities (e.g., walking) tend to amplify both promotional and inhibitory effects of the factors. (3) Some influencing factors display typical non-linear effects, consistent with findings from micro-scale studies.

**Discussion:**

The findings provide comprehensive theoretical support for understanding and optimizing physical activity among urban residents. Based on these results, the study proposes guideline-based macro-level intervention strategies aimed at improving urban physical activity through effective public resource allocation. These strategies can assist policymakers in developing more scientific and targeted approaches to promote physical activity.

## 1 Introduction

Physical activity **(PA)** has been widely recognized as a cornerstone for maintaining and promoting health. Regular PA reduces the risk of non-communicable diseases and offers numerous benefits for maintaining both physical and mental well-being (1). The global lack of PA has become a pressing issue in the field of public health, leading to severe health, economic, environmental, and social consequences (2, 3). To develop targeted interventions that enhance public participation in PA, extensive research is being conducted on the factors and determinants influencing PA (4). These studies span multiple disciplines, including sociology, geography, urban planning, and behavioral sciences (5).

First, the natural geographical environment **(GE)** can impact health by either promoting or inhibiting PA (6, 7). Factors such as season, sunlight duration, atmospheric pressure, wind speed, precipitation, and air quality all have significant effects on PA. Meanwhile, the socio-economic environment **(SE)** has a more complex influence on PA. Individual characteristics (age, gender, income, and education level), social support (from family, friends, and community), and social cohesion have been shown to universally affect all study populations. More importantly, the built environment **(BE)**, as the spatial carrier of public PA, is influenced by the GE while also closely interacting with the SE, has become a key focus of PA influence studies and a crucial intervention tool for promoting PA. Numerous studies have explored the impact of BE factors, including the “5D” variables, landscape, streetscapes, and the visual quality of walking environment (8, 9).

It is worth noting that, despite the extensive research conducted by many scholars on the mechanisms of these three types of influencing factors, most studies tend to limit their variable selection to a particular aspect of these factors while few studies comprehensively consider GE, SE and BE factors together. These three factors, as key determinants of PA, are interconnected and embedded within a complex system (5, 10), jointly shaping public PA behaviors. Obviously, explaining the formation mechanisms from a single perspective has flaws in terms of universality and constrains policymakers in formulating appropriate intervention measures.

The way to obtain data is constrained, which may be one of the reasons behind the aforementioned phenomena. Based on the methods of data acquisition, existing research can generally be divided into two categories: those based on **“small data”** and those based on **“big data”** (9). In terms of PA metrics, traditional “small data” methods primarily involve tracking and recording small samples through surveys and activity logs. For influencing factors, relevant variables are often obtained through field research, questionnaires, and expert evaluations. Although the “small data” approach can capture more detailed, multidimensional information about individuals—such as PA preferences, frequency, intensity, and social support—it is limited by high costs and time consumption, which constrain its use for larger-scale causal analyses. Compared to “small data”, “big data” offers a research paradigm capable of measuring human activities and environmental features at large scales and fine-grained levels. With advancements in information technology, the emergence of volunteered geographic information **(VGI)** has opened up new avenues for PA research. GPS trajectory data recorded by mobile applications, such as Strava in the U.S. and Edooon App or Keep App in China, now enable the collection of vast amounts of PA data at low cost. Besides, a wealth of volunteered urban geographic information, including POI data, street-view imagery, and weather information from remote sensing images and commercial or volunteer-generated online maps, as well as increasingly comprehensive socioeconomic statistics, provide essential conditions for conducting multidimensional research on PA at the macro level.

The second reason lies in the disciplinary differences among scholars, which are primarily reflected in the nature of the “basic units” of their research objects. Research on BE factors mainly comes from the fields of geography and urban planning, where spatial scale is an essential issue. Most existing studies select entire cities or regions as the research object and use community scale or street-level scale as the basic unit of analysis (11). Previous studies have shown that BE factors at multiple scales (community and street level) can promote PA, with different BE characteristics meeting the needs of various levels of activity. In contrast, research on GE factors and SE factors is often produced by scholars in sociology, kinesiology, medicine, and even economics, where spatial scale is less prominent. For these disciplines, the basic unit of analysis is typically based on “population group”. Compared to spatial scale, these studies tend to focus more on the group characteristics of the subjects, often conducting long-term or cross-sectional surveys and statistical analyses of specific populations, such as adolescents, women, the older adult, chronic disease patients, and occupational groups. Although many past studies have been conducted at highly macro spatial scales (such as national or regional levels), the significance of these scales is more related to defining the research subjects—primarily people engaging in PA. Examples include Canadian adolescents (12), Taiwanese adults (13), and farm workers in California, United States (14).

Disciplinary differences lead to inconsistencies in research objects or scales, limiting the development of comprehensive studies. Thus, selecting an appropriate basic unit of research has become a critical issue that needs to be addressed. In this context, cities emerge as a notably rational research unit. Cities are not only the primary areas for PA, reflecting complex socioeconomic structures and built environments, but their diversity and complexity also allow for effective capture of interactions among multiple factors. Additionally, data at the urban level are highly accessible and applicable, enabling comprehensive analyses of various influencing factors. This perspective is supported by a study on the influencing factors of leisure physical activity across 742 cities in China (15).

Returning to PA, frequency, duration, intensity, and type are the main indicators for evaluating its effectiveness (4, 16). Among various PA, mobile activities like running, walking, and cycling are particularly notable. On one hand, these activities require relatively low investment costs in facilities and implementation, making them the most widely practiced forms of PA among the public and a key focus for promoting urban public exercise facilities (17). On the other hand, the emergence of numerous fitness apps provides advantages such as low data acquisition costs and accurate geographic positioning, enabling researchers to conduct joint analyses using large sample spatial data and other geographic information (18). Therefore, this study focuses on mobile PA represented by running, walking, and cycling.

In existing studies on mobile physical activities based on VGI data, common methods for measuring intensity and frequency include: (1) Using street segments, city parks, and other spatial units as the basic unit and generating buffers, then summing the total length of PA paths within the buffer zone and dividing it by the area of the buffer (9, 11, 18, 19); (2) converting VGI data on PA into heat maps to measure the efficiency of PA usage in different regions (8, 18, 20). These methods can be used to obtain the relative intensity of PA within a specific urban area, helping to analyze the impact of the physical environment on PA in different parts of a city. However, these are all relative measures (essentially ratios of PA intensity to urban physical space indicators), making it difficult to conduct cross-city comparisons.

At the PA type level, a 2024 review paper examined 31 sample studies and found that 6 studies treated various types of mobile PA as a whole, 21 focused on a specific type of PA (including 8 on cycling, 10 on running, and 3 on walking), and only 4 studies conducted comparative research on multiple types of PA, exploring differences in how physical environmental factors affect different types of PA (18). Notably, all four studies comparing multiple types of PA were limited to a single city, leaving it unclear to what extent the findings from one city apply to others (9, 19). A study in Bogotá, Colombia, has already shown that research results from developed countries may not be entirely applicable to developing countries (21). Therefore, extracting indicators suitable for cross-city comparisons based on appropriate data sources and sound research design is a necessary prerequisite for comprehensive studies on the mechanisms influencing PA across cities. Finally, this study uses **PA willingness** and **PA intensity** as indicators for conducting horizontal comparisons across cities: (1) **PA willingness**: Refers to the annual number of regular mobile physical activity sessions per capita, reflecting the public’s willingness to actively participate in regular exercise; (2) **PA intensity**: Refers to the average travel distance per session of regular mobile physical activity, indicating the intensity and duration of public exercise behavior.

In summary, to address the aforementioned issues, this study uses cities as the basic research unit and VGI data as the foundation, combined with multi-source urban data, to explore how environmental factors influence both the willingness to engage in and the intensity of mobile PA. Specifically, this study aims to answer the following questions: (1) How can we construct a comprehensive and interpretable research framework based on previous studies, with a focus on solving the challenge of designing indicators for environmental factors across various levels within the urban spatial scale? (2) What is the importance ranking of GE, SE, and BE factors in shaping the willingness and intensity of different types of mobile PA, and how can these be explained?

As a result, this study innovatively combines machine learning methods to construct an interpretable macro-scale framework for mobile PA attribution analysis. We systematically analyze the impact mechanisms of three factors—SE, BE, and GE—on mobile PA, and further provide an in-depth explanation of the results through a comparative analysis with conclusions from micro-scale and small sample studies. Additionally, this study draws on both Western and Eastern research findings and practical cases to propose guideline-based policy recommendations for promoting urban PA from a macro perspective. Through this comprehensive analytical framework, the study not only fills a gap in the current literature but also provides theoretical foundations and practical guidance for urban planning and public health policy development.

## 2 Materials and methods

### 2.1 Framework

**Figure 1:**
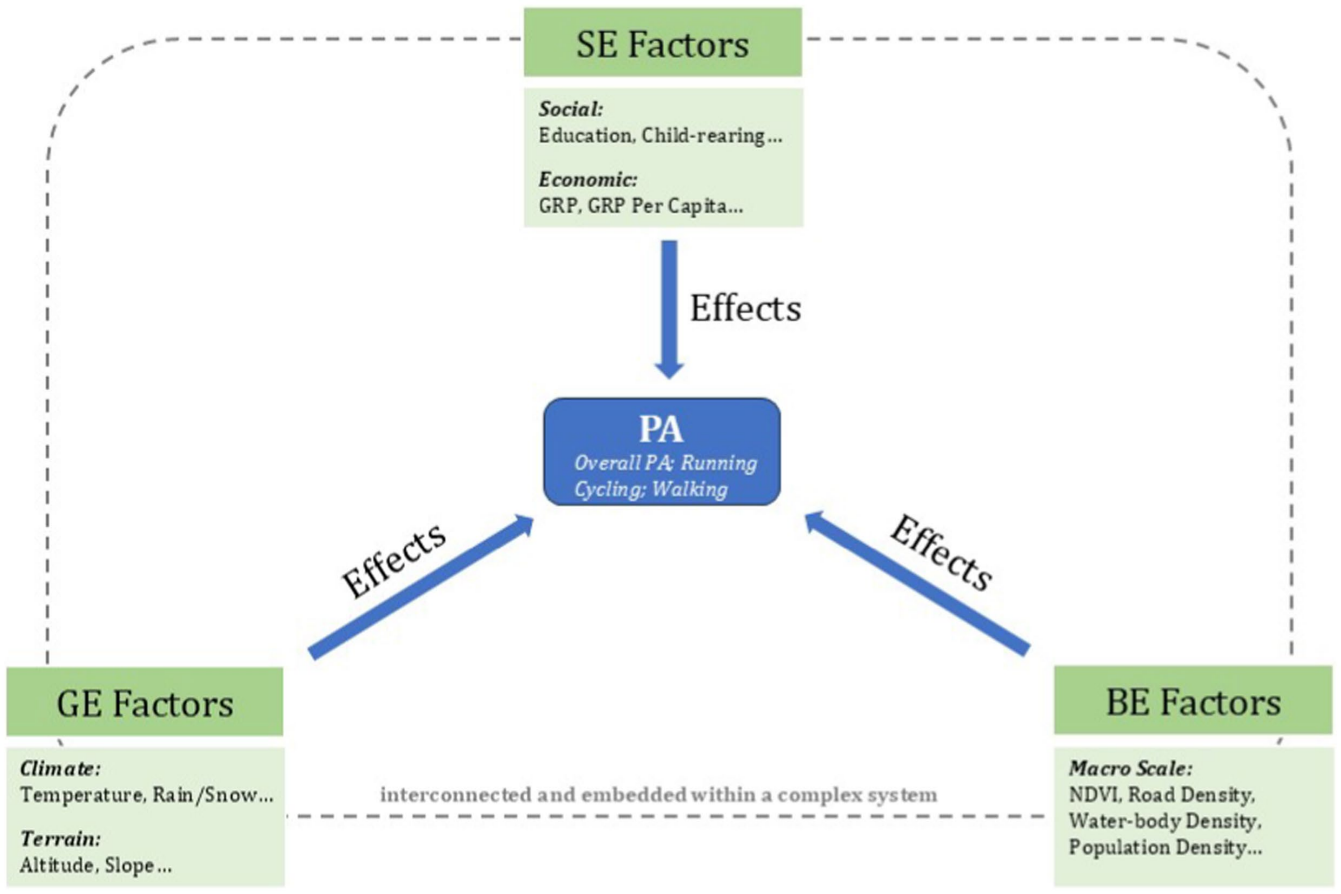
Theoretical framework of this work.

Based on the empirical findings and discussions from the literature review in the Introduction section, this study develops a theoretical framework (Figure 1) to guide the empirical analysis. This framework examines the mechanisms through which the macro environment influences PA from three key dimensions: GE, SE and BE.

**Figure 2:**
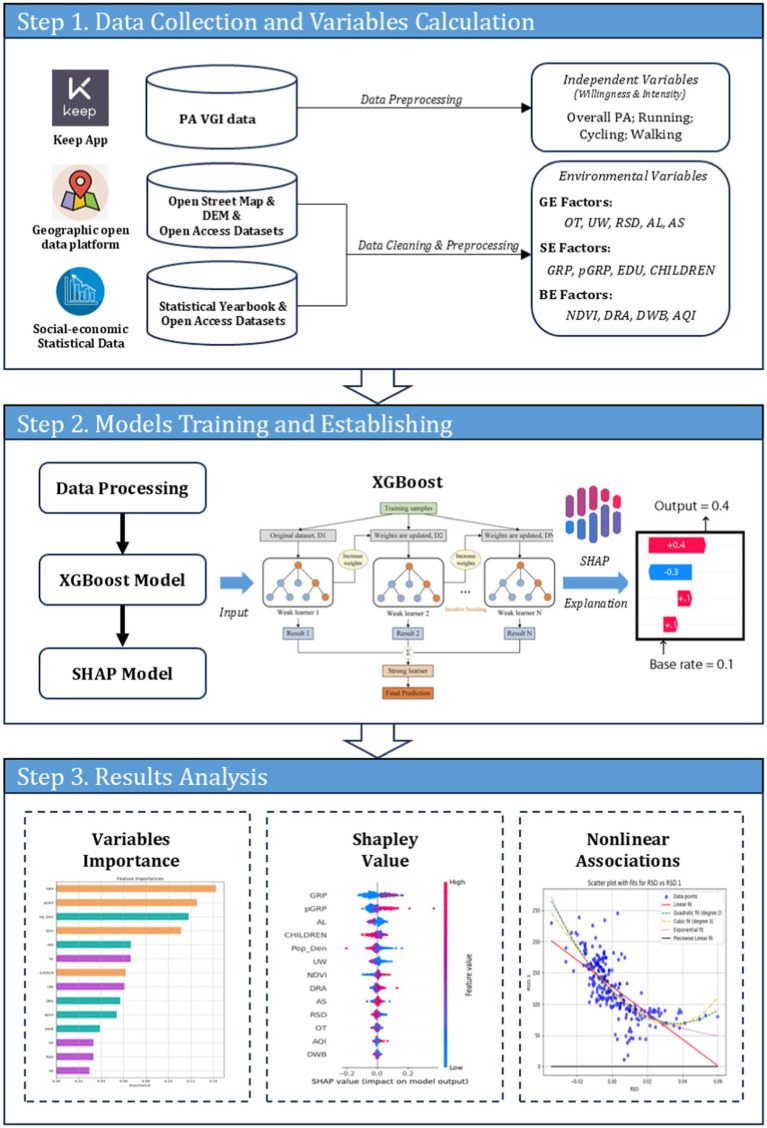
The proposed analytical framework in this work.

This analytical framework, as shown in Figure 2, consists of three main steps: collecting data and calculating variables, training and developing the model, and analyzing and interpreting the results based on variable importance and Shapley values. The analysis focuses on the factors influencing PA willingness and intensity, as well as the nonlinear effects of certain variables.

### 2.2 Study area

**Figure 3:**
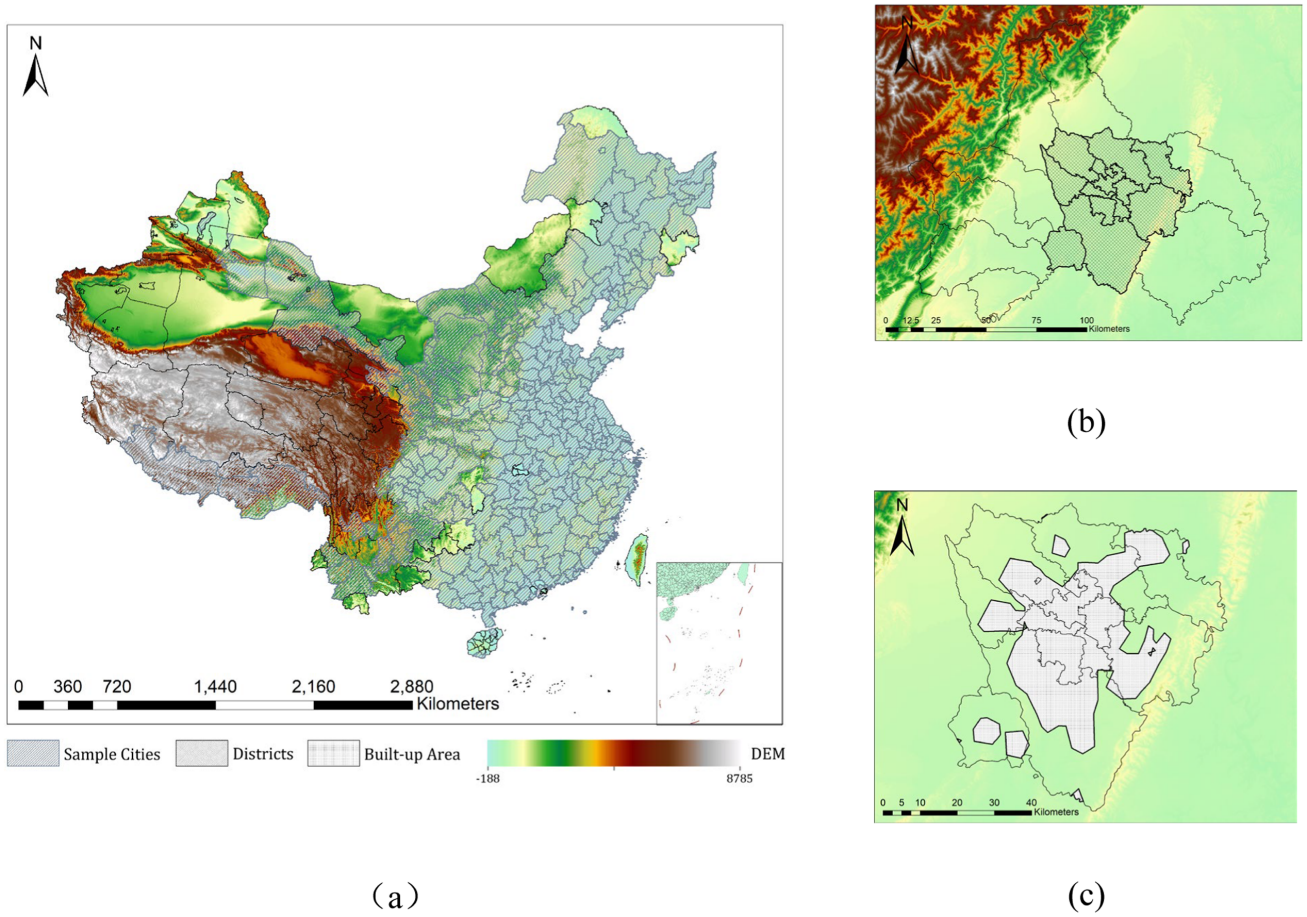
The study area of this work (taking Chengdu as an example shows the study area of every sample city): **(A)** 290 sample cities in China; **(B)** The districts and county-level cities of Chengdu; **(C)** Built-up area in the districts of Chengdu.

This study selects mainland China as the basic research area. Due to its vast territory, the selected sample cities exhibit significant gradient differences in climate, topography, and socioeconomic development levels, providing an appropriate sample set for this study. The research subjects are shown in Figure 3.

China’s urban administrative divisions are categorized into four levels: provincial-level cities *(Zhixiashi)*, prefecture-level cities *(Dijishi)*, county-level cities *(Xianjishi and Xian)*, and towns *(Zhen) (Ministry of Civil Affairs, 2010)*. Prefecture-level cities typically govern several districts and county-level cities. For example, Chengdu, the capital of Sichuan Province, administers 12 districts and 8 county-level cities. Considering data availability and consistency in statistical standards, we selected 290 prefecture-level cities in China as the study objects, focusing on all districts within these cities as the administrative boundaries for our research. This is justified because urban districts are the primary areas for economic activities, and their statistical data effectively reflect the economic, environmental, and social development status of cities. In the annual China Urban Statistical Yearbook published by the National Bureau of Statistics of China, urban districts are treated as separate statistical units for the purpose of collecting social and economic indicators.

It is important to note that even we take highly urbanized districts as the administrative boundaries for the study, there are still certain issues. Within the administrative borders of each urban district, there exists a mixture of urban, suburban, and rural areas, as well as significant agricultural land and sparsely populated forest and mountainous regions. Clearly, these areas should not be considered as spatial objects for studying public PA. Moreover, including these regions—characterized by high greenery coverage (farmland, forests, etc.) and substantial variations in elevation and slope (mountains)—would distort indicators such as vegetation and water coverage, average elevation, and slope in the final calculations for each city. Therefore, we ultimately selected the built-up areas within the sampled districts as the final spatial scope for our research. According to the official definition by China’s Ministry of Housing and Urban–Rural Development, built-up areas refer to regions within urban boundaries that possess sufficient municipal and public infrastructure, typically surveyed and mapped by provincial-level governments *(Ministry of Housing and Urban–Rural Development, 1998)*. Unfortunately, maps of these built-up areas are not publicly available. Some scholars have identified urban built-up areas using open-source data, such as remote sensing imagery (22), nighttime light data (23, 24), or point-of-interest markers provided by commercial mapping companies (25). In this study, the identification of built-up area boundaries relied on the findings of these scholars, which will be detailed in the subsequent Datasets section.

### 2.3 Datasets

#### 2.3.1 PA data

Users’ PA data is recorded by the Keep App and does not involve personal privacy. Keep App, along with Codoon App and Yuepaoquan App, is one of the three most popular outdoor fitness tracking apps in China, boasting a large user base. According to the “Analysis Report on the Development Prospects and Investment Strategy Planning of China’s Sports and Fitness Apps” published by the Zhiyanzhan Consulting Industry Research Institute, at of the end of 2022, Keep App held a 32.5% market share, ranking first in China’s sports and fitness app industry.

The popular routes in Keep App^1^ are user-generated and shared by users who consider them to be safe and publicly accessible, and high-quality routes in urban space. These routes represent a collection of locations suitable for mobile PA such as running, walking, and cycling. The route information includes a unique route ID (assigned by the system upon creation), route name, geographical location (latitude and longitude coordinates of the route’s starting point), site type, route length, number of times the route has been completed, creation date, and activity type proportions (including running, cycling, and walking). Users can view nearby popular routes in Keep App and explore popular routes in different areas by adjusting the map position or zooming in and out on the map interface.

It is important to note that the route data used in this study differs from the commonly utilized route data in previous research. As mentioned in the Introduction, the PA datasets used for calculating buffers or heat maps typically encompass all PA that occurred within the study area, which may include a significant number of commuting-related activities (many individuals open fitness apps to track their walking or cycling routes during commutes). In contrast, the data used in this study consists of check-in data for fixed exercise routes, primarily aimed at regular workouts. This can be viewed as a dataset reflecting public patterns of PA, better capturing individuals’ environmental preferences for PA. The reasoning behind this is that commuting-related PA often prioritize time costs over environmental preferences when selecting routes. Additionally, commuting behaviors, such as those related to going to and from work, are less influenced by climatic conditions, making it more challenging to assess the sensitivity of PA to GE factors.

To avoid omission as much as possible in the process of acquiring route data, we utilized ArcGIS software to create a grid with 1,000-meter square cells within the study area. After obtaining the coordinates of the grid’s center points, we retrieved all route information within a 2,000-meter radius. Ultimately, we identified 221,451 popular routes within the built-up areas of 290 prefecture-level cities in China. After deduplicating the data using route-ID as a unique identifier, we finalized the dataset with 63,819 popular routes.

#### 2.3.2 Multi-source urban data

The multi-city dataset primarily includes urban built-up area boundary, Normalized Difference Vegetation Index (NDVI), water body, population data, elevation (DEM), weather and socioeconomic data, and weather data. The urban built-up area and NDVI datasets are publicly available datasets published by scholars online. Water body is sourced from OpenStreetMap.^2^ Population comes from ***the 7th national population census*** conducted by the National Bureau of Statistics of China, with a precision at the district level. Elevation is obtained from the Geospatial Data Cloud^3^, a geographic big data platform established by the Computer Network Information Center of the Chinese Academy of Sciences, which provides search, retrieval, storage, and visualization services for geospatial data. The SRTM DEM UTM 90 M data product is utilized in this study. SE data is derived from the ***China City Statistical Yearbook*** published by the National Bureau of Statistics of China. Weather is sourced from 2,345 Weather Network.^4^ Data on average years of education is obtained from Macro Data Network.^5^ It is important to note that not all environmental variable data are raw data as mentioned above; some are derived from calculations based on the raw data, with detailed methodologies discussed in the variable explanation section. All data represent cross-sectional data for China in 2020. Data source descriptions are presented in Table 1.

**Table 1:** Data source descriptions.

Data	Source
Built-up area boundary	Open Access Dataset (83)
NDVI	Open Access Dataset (84)
Water-body data	Open Street Map (www.openstreetmap.org)
Population	China’s 7th national population census
DEM	Geospatial Data Cloud (www.gscloud.cn)
Socioeconomic data	China City Statistical Yearbook
Weather data	Captured from internet (waptianqi.2345.com)
Education data	Macro Data Network (www.macrodatas.cn)

### 2.4 Variables

#### 2.4.1 Dependent variables

We use the annual average number of times per person participating in PA in a sample city to reflect the public’s willingness to engage in regular mobile PA. The average distance per PA session per person represents the general intensity of PA in that sample city. It is important to note that while Keep App holds a significant market share in China and has a large user base to support nationwide analysis, user tool choices may introduce potential biases in our dataset. This issue seems unavoidable in research utilizing VGI data (15). Therefore, to minimize this bias when calculating per capita values, we introduced the concept of the potential user population.

For example, in China, minors under the age of 15 (as compulsory education stage in China generally concludes around age 15) are not allowed to privately own mobile phones due to academic restrictions, and they are often scheduled for organized PA in school to promote health, making it unlikely for them to be consistent Keep App users. Additionally, according to previous studies, users of Keep App under the age of 25 years account for 25%, those 25–35 years old account for 59%, those 35–40 years old account for 13%, and those over 40 years old account for 3% (26). Therefore, we define the total population between the ages of 15 and 40 as the potential user base for each city.

For the time dimension, we calculated the total number of months since the route was created by taking the difference between the data collection date (June 2024) and the route creation date. Using route length, cumulative check-in counts, and activity type proportions directly obtained from the raw data, we calculated overall PA willingness *(W-overall)* (Equation 1), willingness by PA type *(W-type)* (Equation 2), overall PA intensity *(I-overall)* (Equation 3), and PA intensity by type *(I-type)* (Equation 4). The formulas are as follows:


(1) $$W-\mathrm{over}\mathrm{all}=\frac{1}{P}\overset{n}{\underset{i=1}{\sum }}\left(\frac{Mi}{Ti}\times 12\right)$$

(2) $$W-\mathrm{type}=\frac{1}{P}\overset{n}{\underset{i=1}{\sum }}\left(\frac{Mi\times Ri}{Ti}\times 12\right)$$

(3) $$I-\mathrm{overall}=\frac{\sum_{i=1}^{n}\left(Li\times \frac{Mi}{Ti}\times 12\right)}{\sum_{i=1}^{n}\left(\frac{Mi}{Ti}\times 12\right)}$$

(4) $$I-\mathrm{type}=\frac{\sum_{i=1}^{n}\left(Li\times \frac{Mi\times Ri}{Ti}\times 12\right)}{\sum_{i=1}^{n}\left(\frac{Mi\times Ri}{Ti}\times 12\right)}$$

In this context, P represents the potential user population of the sample, Ti represents the cumulative number of months from the route’s creation to the data collection date, Mi represents the cumulative number of check-ins since the route’s establishment up to the time of data collection, Li represents the route length, and Ri represents the proportion of check-ins for a specific type of activity on the route. Descriptive statistics for the dependent variables are presented in Table 2.

**Table 2:** Descriptive statistics of independent variables.

Variables	Units	Mean	Max	Min	Std. Dev
W-overall	Person-times	0.35	1.22	0.02	0.21
W-running		0.30	1.00	0.01	0.18
W-cycling		0.01	0.08	0.00	0.01
W-walking		0.04	0.20	0.00	0.03
I-overall	Meters	1488.67	6041.31	734.37	602.84
I-running		1357.38	6392.53	681.68	619.23
I-cycling		4545.51	17388.36	1900.54	1993.15
I-walking		1520.05	3541.50	764.64	422.69

#### 2.4.2 Environmental variables

The environmental variables include natural geography, socioeconomic factors, and built environment factors. Since no comprehensive systematic studies have previously integrated these three levels and the spatial scale of the research object differs significantly from existing literature, the design of indicators is a key focus of this paper. Environmental variables’ descriptions are presented in Table 3.

**Table 3:** Descriptive statistics of environmental variables.

Dimensions	Variables	Units	Mean	Max	Min	Std. Dev
GE factors	Optimal exercise temperature *(OT)*	Days	180.70	365	80	34.48
	Unsuitable Wind Force-Scale for Exercise *(UW)*	Days	20.10	154	0	27.37
	Rainy/Snowy Days (*RSD)*	Days	124.89	245	11	43.49
	Altitude *(AL)*	Meters	405.59	4531.44	1.50	680.15
	Appropriate Slope for-Exercise *(AS)*	%	86.91	100.00	15.46	16.51
SE factors	Gross Regional Product *(GRP)*	10000CNY	210.90*10^5^	3870.10*10^5^	3.80*10^5^	454.45*10^5^
	*Per Capita* GRP *(pGRP)*	CNY	74219.73	180871.00	24353.00	34245.99
	Average Years of Schooling *(EDU)*	Years	9.27	12.21	4.81	0.88
	Percentage of Population-aged 0–5 *(CHILDREN)*	%	5.34	10.00	2.01	1.36
BE factors	Normalized Difference Vegetation Index *(NDVI)*	%	29.44	47.19	14.15	4.95
	Density of Road Area *(DRA)*	%	15.34	31.78	2.00	4.82
	Density of Water-body Buffer-zone *(DWB)*	%	27.00	77.11	1.85	12.45
	Density of Population *(Pop_Den)*	Persons/km^2^	12566.08	58634.97	3138.77	5305.42
	Days with Air Pollution *(AQI)*	Days	9.96	56	0	12.04

##### 2.4.2.1 GE variables

Based on the previous discussion, GE variables such as seasonality, temperature, light exposure, atmospheric pressure, wind force scale, precipitation, and air quality significantly influence PA. Due to data availability, we selected variables from climate and terrain dimensions, including temperature, wind force scale, elevation, precipitation, and slope. Given the popularity of evening running and walking after dinner in China, we excluded light exposure from the indicators. Since atmospheric pressure is strongly correlated with elevation and the latter can reflect oxygen levels—which significantly impact PA—we opted for elevation as a more suitable substitute. Additionally, because air quality is closely linked to urban development and management levels, it was included in the built environment variables. Unfortunately, we could not consider seasonal factors due to the unavailability of time-series data on PA. We have attempted to address this issue through the following research design:

It’s worth noting that in previous studies, temperature, precipitation, slope, and wind force scale were often used as variables based on their average values. While this approach is reasonable, it presents certain limitations. Averages can indeed reflect the general level of a given variable, but they fail to capture its variability or extremity. For instance, two cities at the same latitude or within the same climate zone (e.g., one inland and one coastal) may have similar average temperatures, but the likelihood of extreme temperatures may differ significantly. What influences people’s decision to engage in outdoor PA is the specific temperature at a given time, not the annual average.

Thus, we designed our indicators with a focus on geographic and climatic conditions favorable to PA, including: (1) Number of days with suitable exercise temperatures (number of days per year with temperatures between 15°C and 25°C); (2) Number of days with unsuitable wind conditions (number of days with wind speeds of ≥4 on the Beaufort scale); (3) Number of non-rain/snow days (number of days per year with rainfall or snowfall exceeding light rain or light snow); (4) Proportion of suitable exercise slope (percentage of areas with slopes ≤10% within the city’s built-up areas); and (5) Average elevation. This approach allows us to better reflect the specific conditions that directly impact PA rather than relying solely on average values.

##### 2.4.2.2 SE variables

In the SE variables, individual circumstances, social support, and social cohesion are three main factors influencing PA. In the previously mentioned “small data” research paradigm, individual circumstances including gender, age, income, and education level have shown significant effects on PA research. However, this may not be the case in the “big data” research paradigm. Studies have indicated that there is a notable urban–rural disparity in age structure in China, and in the past 30 years of intense urbanization, the issue of left-behind older adult and children in rural areas has become a hot topic in academia and society. However, there is no evidence to suggest that such age structure differences exist among urban areas. Similarly, there is no definitive evidence proving significant gender differences between cities in China.

Considering the availability of data, we selected GDP per capita and average years of education as indicators at the individual level. In past research, social support primarily reflects the support of individuals’ families and communities for engaging in PA. While this can be obtained through tracking surveys in micro-level studies, it becomes difficult to measure family structure and community support with a unified indicator on a macro scale. Given that the data source mainly consists of users aged 15 to 40, who encompass a large portion of the marriageable and childbearing population in cities, raising young children and caring for older adult individuals may take up a significant amount of their leisure time. Infants and toddlers require parental supervision and companionship, which may limit parents’ opportunities for regular PA. Therefore, we calculated the ratio of children aged 0–5 to the user population as an indicator.

It’s important to note that accompanying children and older adult individuals during activities like walking or playing can somewhat promote adults’ PA. However, due to the unpredictability of children’s and older adult people’s activities (they may stop somewhere at any time such as parks or open spaces to play or rest), it is rare for caregivers to record their PA data on an app during these companionship (as their walking or jogging can be interrupted at any moment). Therefore, we found it challenging to capture this promoting effect in our existing data, and thus did not consider indicators for older children or older adult populations.

Finally, social cohesion generally appears in cross-regional or cross-national studies to reflect the impact of different cultural backgrounds and economic development levels on the amount and type of PA among the public. At the level of economic development, we chose GDP to measure the economic development level of different samples. In terms of cultural background, since this study does not involve cross-regional or cross-national research, although ethnic and cultural differences in China may lead to some variation in the types of PA (such as the popularity of lion dancing and dragon boat racing in southern China), these differences can be overlooked for the three specific activities of running, cycling, and walking considered in this paper. In summary, the selected SE variables for this study include GDP, GDP per capita, average years of education, and the proportion of the user population that consists of children aged 0–5.

##### 2.4.2.3 BE variables

Regarding BE variables, the most commonly used model in existing literature for assessing the built environment is the 3D (or 5D and 7D) model. This model was initially proposed by Cervero and Kockelman in 1997, highlighting that the built environment can be evaluated through density, diversity, and design (27). Ewing and Cervero later expanded the “3D” model into the “5D” model (adding destination accessibility and distance to transit) and the “7D” model (further incorporating demand management and demographics) (28). Factors such as service facility accessibility, population density, road intersection density, landscape characteristics, and visual quality have all been shown to directly influence PA.

In this study, we calculated the population density of each city using data from China’s seventh national census and the built-up area data from the China Urban Statistical Yearbook. We computed road area density by dividing the road area by the built-up area, based on data from the China Urban Statistical Yearbook, to represent road density and service facility accessibility.

As for landscape characteristics and visual quality, previous research on the relationship between PA and BE has often been conducted on a micro-scale. While the visual quality represented by street-view images has been shown to have a significant impact on PA, this indicator becomes difficult to quantify on an urban scale. This is because, on the one hand, differences in infrastructure across cities and variations in street-view image angles and equipment result in inconsistent green view index standards. On the other hand, street-view images are typically provided by map service companies, and due to differences in commercial value, the investment in capturing street views varies between cities, leading to significant differences in the density and frequency of street-view image collection across cities. These factors make it difficult to use street-view images to measure cross-city differences.

Therefore, we integrated landscape characteristics and visual quality into a single indicator, using the city’s NDVI index and water body coverage area density to measure these aspects. For water body coverage area density, considering that our PA data reflects regular mobile PA, which tend to have a strong “proximity” characteristic, it is unlikely that people would regularly drive or take public transportation to destinations more than 15 or 20 min away for running, cycling, or walking and then return home. Thus, in calculating water body coverage area density, we used water body data from OpenStreetMap and applied a block-level (250 m) buffer in ArcGIS to calculate the ratio of the buffer area to the city’s built-up area.

Finally, we selected the number of days in a year with moderate or higher levels of air pollution as an indicator to evaluate the air quality of each sample city. The final BE indicator set includes population density, road area density, water body coverage area density, city air quality, and the average NDVI index of each city.

### 2.5 Methods

#### 2.5.1 XGBoost model

Machine learning (ML) has reshaped the way we understand and analyze the intricate relationships among variables. Methods like Random Forest (RF) and eXtreme Gradient Boosting (XGBoost) have proven effective in handling multi-source data and revealing intricate connections between behavior and the environment (9, 11, 29–33). GBDT model (11, 29, 34), Random Forest model (9, 33), and XGBoost model (35) have shown excellent performance in exploring the nonlinear impacts of urban BE on various types of PA, including active transportation (32), children’s PA (35), and walking or cycling (36, 37).

Given the lower interpretability of ML methods, some literature has introduced variable importance ranking, partial dependence plots, and SHAP models to improve global or local explanations (38, 39). The XGBoost model employs a gradient boosting approach, successively fusing decision trees with gradient descent to reduce prediction errors (40). Research shows that a well-calibrated XGBoost model generally surpasses alternatives—such as Random Forest or neural networks—in tackling supervised learning tasks. Plus, XGBoost’s compatibility with SHAP facilitates the accurate calculation of Shapley values using the Tree SHAP algorithm (41).

#### 2.5.2 SHAP model

Although ML is widely used in fields like geography, it is often seen as a “black box” model, where the processes behind the model’s predictions are not fully understood. In geography and urban planning, however, it’s crucial to interpret these models, as researchers want to understand the underlying principles behind the data, rather than just making predictions. (31). Without understanding these principles, predictions lose their value.

To improve interpretability in ML, methods like LIME (Local Interpretable Model-agnostic Explanations) and SHAP (Shapley Additive exPlanations) have emerged, offering more detailed and personalized explanations and attributions than traditional global interpretative techniques (42, 43). The SHAP model provides not only global explanations but also local ones (31). It quantifies the impact of each independent variable on the dependent variable, using Shapley values derived from game theory. The formula for the Shapley value of feature *i* is as follows (Equation 5):


(5) $$\emptyset i=\underset{S\subseteq N\left\{i\right\}}{\sum }\frac{|S|!\left(n-|S|-1\right)!}{n!}\left[f\left(S\cup \left\{i\right\}\right)-f\left(S\right)\right]$$

Here, ∅i represents the contribution of feature *i*, *N* denotes the set of features, $f\left(S\cup \left\{i\right\}\right)$ and $f\left(S\right)$ represent the model results with and without feature *i*, respectively.

## 3 Results

### 3.1 Model performance

This study established 8 models, each corresponding to a dependent variable. 80% of all samples were used as the training set, while 20% were used as the test set. The Optuna module in Python was used for hyperparameter tuning, with the highest *R*^2^ value on the test set as the optimization target. A 5-fold cross-validation was applied to prevent overfitting. After 20,000 rounds of tuning for each model, the optimal parameters were obtained. *R*^2^ and Mean Squared Error (MSE) were used as the evaluation metrics for model performance. It is important to note that the absolute value of MSE is significantly related to the magnitude of the dependent variables, leading to large differences in MSE across different models. The performance of all models is presented in Table 4.

**Table 4:** Model performance of 8 models.

	W-overall_Model	W-running_Model	W-cycling_Model	W-walking_Model
*R* ^2^	0.5711	0.5570	0.3372	0.6509
MSE	0.0226	0.0170	0.0002	0.0004

	I-overall_Model	I-running_Model	I-cycling_Model	I-walking_Model
*R* ^2^	0.3781	0.4224	−0.0362	0.2242
MSE	409902.0069	360018.6788	55476898.8559	139211.7681

Following the common practices in existing studies (9, 32, 33, 44, 45), we compared the predictive performance of the selected XGBoost model with commonly used models, including Random Forest (RF), Gradient Boosting Decision Tree (GBDT), and Ordinary Least Squares (OLS), on the sample dataset to ensure the accuracy of model selection. We used *R*^2^ (to evaluate the accuracy of model predictions) and MSE (to assess the difference between predicted and actual values, where the absolute value of MSE is related to the magnitude of values in the dataset) to compare the XGBoost model with RF, GBDT, and OLS.

**Figure 4:**
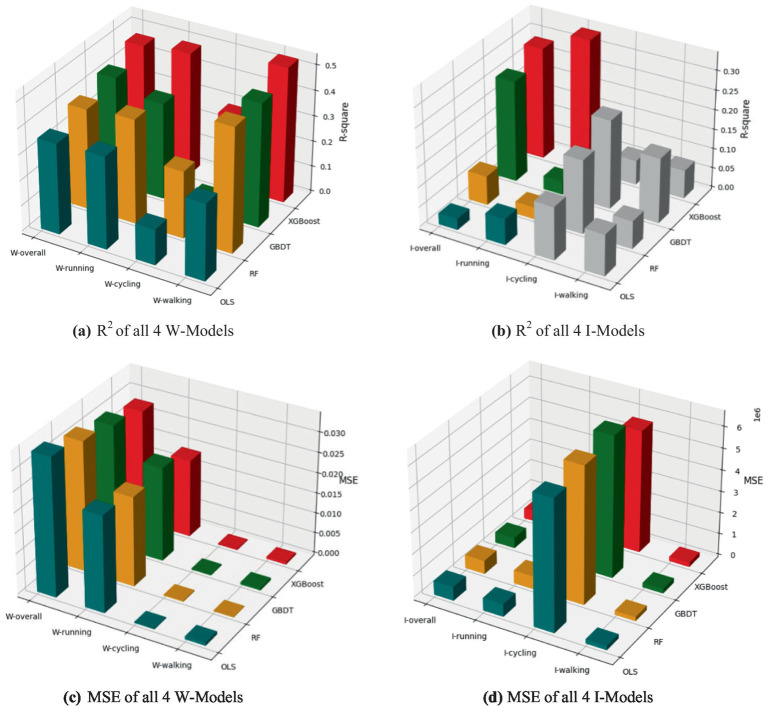
Performance comparison of different models. **(a)** *R*^2^ of all 4 W-Models; **(b)** R² of all 4 I-Models; **(c)** MSE of all 4 W-Models; **(d)** MSE of all 4 I-Models. The gray portions in the bar chart indicate that the model’s *R*^2^ is negative.

The results show that, in the majority of models, the predictive performance (*R*^2^) of the XGBoost model surpasses that of the other models. Except for the I-walking model (where the MSE of the XGBoost model is 0.07, compared to the 0.01–0.05 range for the other three models), the prediction errors of the XGBoost model are consistently lower than those of the other commonly used models. The performance comparison of different models is presented in Figure 4.

Given that the goal of this study, as well as similar research, is not the absolute predictive performance of the model but rather the mechanisms and principles revealed behind the model, it is difficult to establish a single standard for determining model validity. This is due to the complexity of the factors influencing PA behavior, the diversity of urban environments, and the variability in indicator selection across different studies. For instance, it would be inappropriate to consider a model highly reliable solely based on an *R*^2^ value above a certain threshold. Therefore, we referenced the model reliability results from similar studies to assess the reliability of our model. Unfortunately, in more than half of the 10+ machine learning-based studies we reviewed, reliability indicators were not explicitly provided, which posed a challenge for us. Model performance from past works on PA analysis is presented in Table 5.

**Table 5:** Model performance of previous works on PA analysis.

Reference	Influencing factors	Scale	Method	Model performance
Yang et al. (9)	Building environment & Visual-landscape & Socio-economic	TAZ	GW-RF model	*R*^2^: 0.48–0.64
Zhuang et al. (29)	Built-environment & Traffic conditions	Road_buffer (20 m)	GBDT model	*R*^2^: 0.451–0.926
Cheng et al. (30)	Built-environment & Socio-demographics	500 m	RF model	Pseudo-*R*^2^: 0.313
Yang et al. (11)	Macroscale BE & Microscale BE & Socio-economic	TAZ	GBDT model	*R*^2^: 0.926
Zhou et al. (85)	“5D” Built-environment	Road_buffer (20 m, 50 m,100 m,500 m)	RF model	*R*^2^: 0.236–0.327
Liu et al. (32)	Built-environment & Visual-landscape	Block	RF model	*R*^2^: 0.451–0.576
Wang et al. (86)	Built-environment & Social environment	District	XGBoost	*R*^2^: 0.838
Liu et al. (44)	Built-environment & Social-economic	TAZ	XGBoost	*R*^2^: 0.849–0.855

By examining the research scale, variable selection, and final model performance of existing studies, the following conclusions can be drawn:

(1) **Model performance is directly related to the complexity of variables.** Among the four studies with model performance exceeding 0.8, the core research indicators are all micro-scale BE variables. Two of these studies incorporate socioeconomic attributes such as housing prices, rental prices, and GDP—variables that solely reflect local economic development—making the composition of variables relatively simple (11, 46). However, when complex indicators such as individual and household economic conditions, family structure (30), and traffic conditions (29) are added to the variable set, model performance significantly declines.
(2) **Model performance is related to the predictability of the behavior itself.** Compared to physical activity, which exhibits greater randomness, travel behavior is easier to predict. As a result, despite the inclusion of individual-level variables such as education level, housing size, family structure, and car ownership, studies like (44) still achieve high model performance in predicting travel behavior choices.
(3) **Smaller variable differences within single-city samples improve model performance.** As the above studies all use single cities (primarily economically developed regions such as Beijing, Nanjing, and Xiamen) as samples, the relatively small variation in variables across research units within the same sample may also contribute to improved model performance.

Therefore, considering the broader spatial scale of the study, the more complex variable set (including SE, GE and BE factors), and the greater variability in indicators among samples, as well as the increased difficulty in predicting physical activity after excluding commuting behaviors, we believe that if the *R*^2^ performance of the model reaches the general level of similar studies and the results are interpretable, the model can be considered reliable.

In summary, based on the model performance from similar past studies (Table 5) and considering the interpretability of the models in this study, we categorized the models into three levels: reliable, relatively reliable, and unreliable. Among all eight models, those reflecting activity willingness demonstrated higher reliability, including 3 reliable models and 1 relatively reliable model (W-cycling). In contrast, the models reflecting activity intensity showed lower reliability, comprising one reliable model, 2 relatively reliable models (I-overall and I-walking), and 1 unreliable model (I-cycling).

We hypothesize that this is related to the check-in mechanism of the Keep App. For example, when a person runs on a popular 3 km route, whether they complete 80% of the route (or even less) or run the entire route (or more) would still count as a valid check-in. This discrepancy could lead to a divergence between actual activity intensity and the calculated activity intensity.

Additionally, the reliability metrics of the models show that the performance of the cycling behavior model is significantly lower than those for running, walking, and overall activity. We believe this is largely due to the selection of model indicators. The widespread use of shared dockless bicycles in China has greatly boosted public enthusiasm for cycling, whether as a means of transport or as exercise (47). However, the difficulty in acquiring shared dockless bicycle deployment data at a large sample size may have negatively impacted the reliability of this study’s analysis of cycling behavior.

### 3.2 Variables’ importance

**Figure 5:**
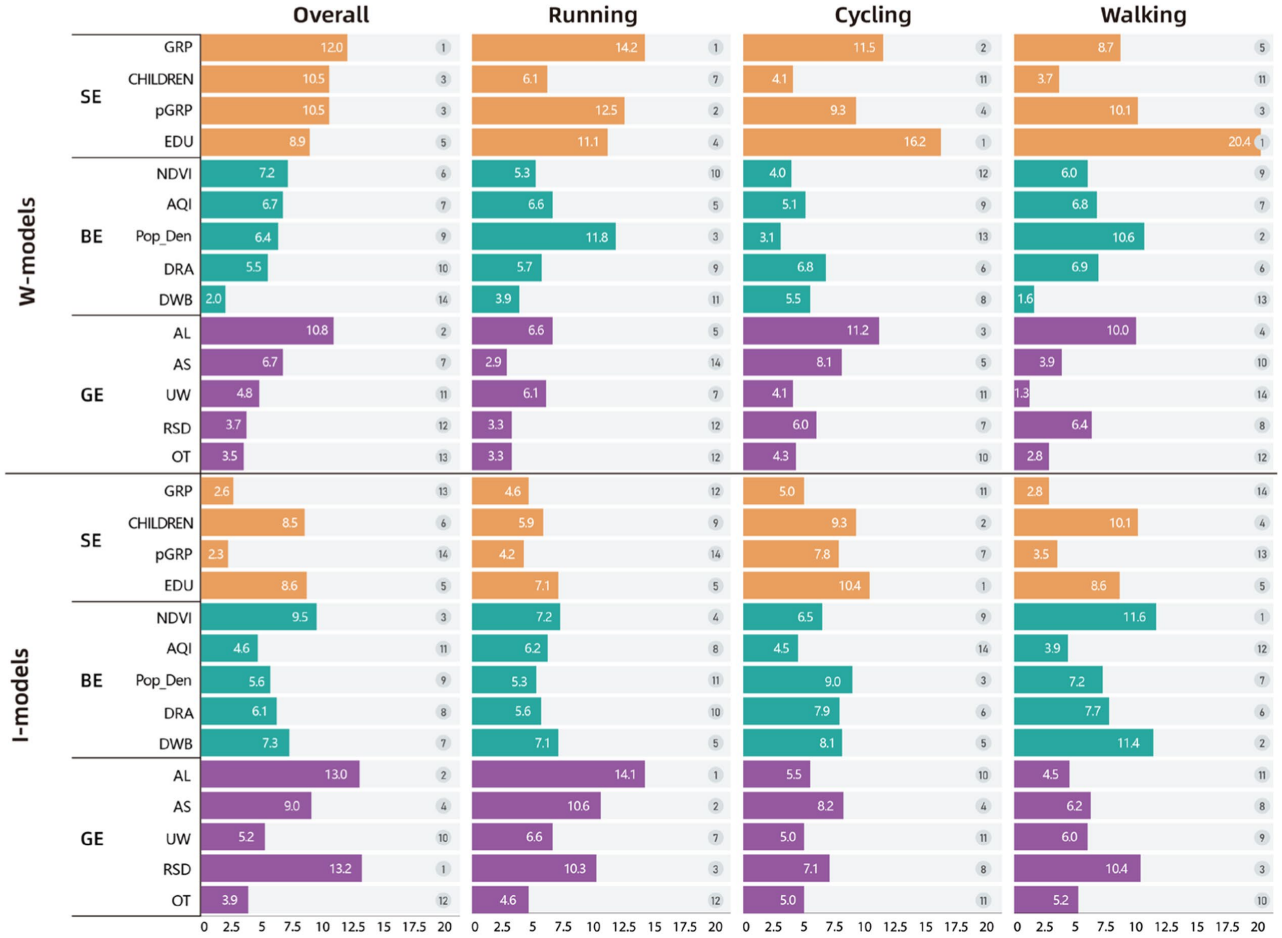
Variables’ importance and ranks of all the models.

**Figure 6:**
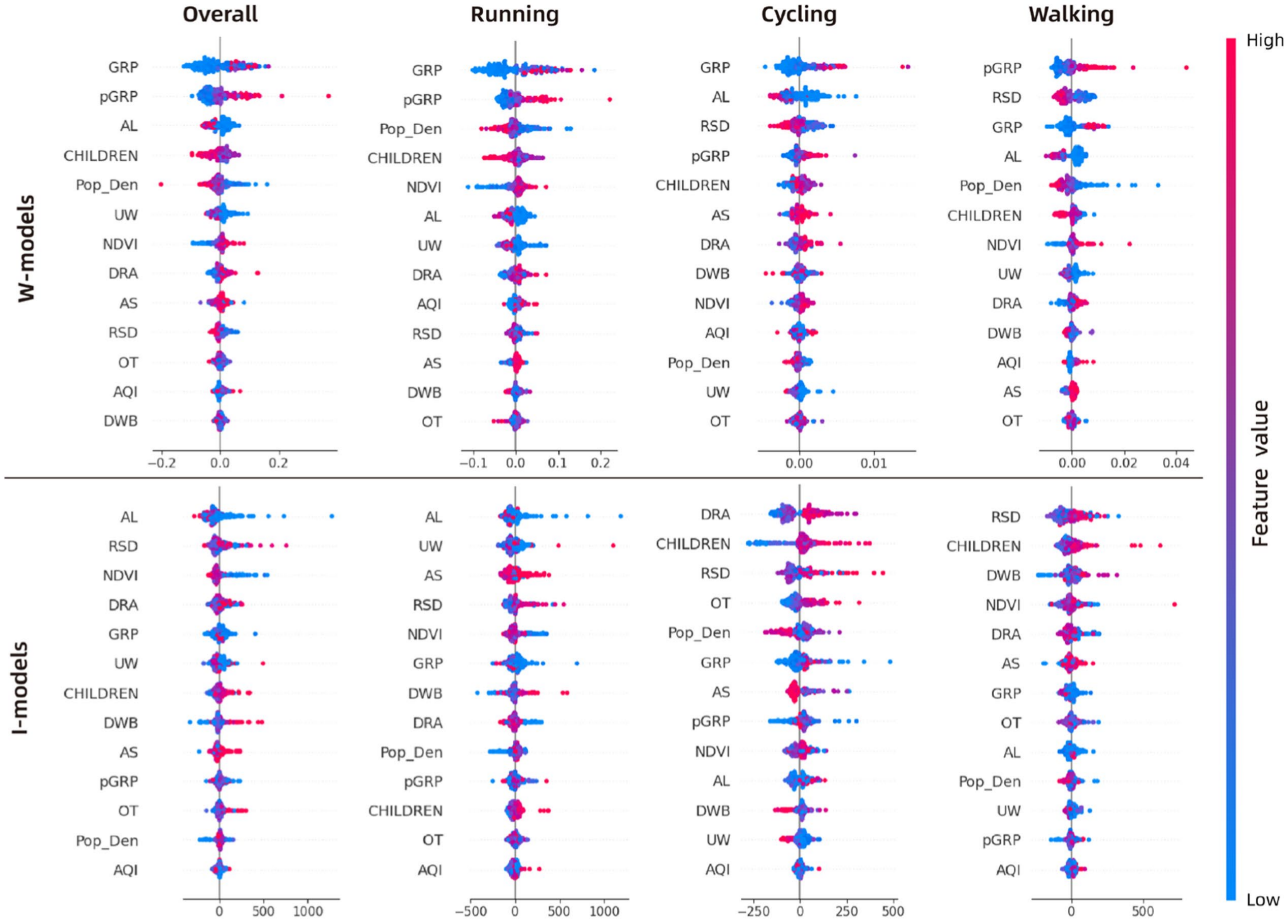
Variables’ SHAP values of all the models. SHAP plots show how an increase or decrease in a specific feature impacts the result (promotive or inhibitory): (1) The X-axis in the image represents the SHAP value of a specific feature (e.g., GRP) for each sample: the magnitude indicates the level of contribution; while the direction—positive or negative—indicates whether the effect is promotive or inhibitory. (2) The color of each sample point represents the actual value of the feature: Red indicates higher actual values; Blue indicates lower actual values.

The contributions of the 14 variables across the eight models were calculated using the average SHAP values (Figure 5). Figure 6 presents the SHAP values and the direction of influence for each variable in each model.

#### 3.2.1 Variables’ importance on the willingness of PA

In terms of PA willingness, the overall model results show that SE factors (with an average variable ranking of 3rd) are the primary drivers of public willingness to engage in PA, while the BE (with an average ranking of 9.2) and GE factors (with an average ranking of 9th) have relatively lower influence on PA willingness.

First, SE factors have the most significant impact on public overall PA willingness. Based on the direction of influence, higher economic levels (GRP contributing 12%, pGRP contributing 10.5%) and education levels (EDU contributing 8.9%) are the main drivers for increasing PA frequency. For specific types of PA, the variable contributions in the running model align closely with the overall model (with the ranking GRP > pGRP > EDU). However, the cycling and walking models differ, with education level rising to the top, contributing 16.2 and 20.4%, respectively. In the overall model, child-rearing emerges as the primary limiting factor for PA willingness, ranking 3rd among all 14 variables with a contribution of 10.5%. However, this effect is less pronounced in specific activity types (ranking 7th in the running model and 11th in both the walking and cycling models).

Second, in the three most reliable models (overall model, running model, and walking model), population density, air quality, NDVI, and road area density exhibit strong influence. However, their effects differ across activity types. In the overall model, these four indicators show a more uniform influence, but differences emerge in running and walking models. Population density stands out as a significant constraint on public activity willingness, contributing 11.8% in the running model and 10.6% in the walking model.

Finally, among GE factors, elevation (AL) is the most significant limiting factor on public PA, showing a strong impact across all models. The influence of suitable slope for PA is also notable, particularly in the overall model (6.7%) and the cycling model (8.1%).

#### 3.2.2 Variables’ importance on the intensity of PA

In terms of PA intensity, the model results differ significantly from those of PA willingness. First, the importance of SE factors declines sharply across the three models being discussed (the cycling model is excluded due to low reliability). In the overall model, SE factors have an average ranking of 9.5; in the running model 10th and in the walking model 9th. On the other hand, the contribution of GE factors increases considerably, with an average ranking of 5.8 in the overall model, 5th in the running model, and 8.2 in the walking model. BE factors show moderate influence in the overall model (average ranking of 7.6) and the running model (average ranking of 7.6), but they have the highest contribution in the walking model (average ranking of 5.6).

Regarding overall activity intensity and running intensity, at the GE level, elevation (with contributions of 13 and 14.1%, respectively) is the primary limiting factor, consistent with the results from the activity willingness model. However, unlike in activity willingness, the number of non-rain/snow days (contributing 13.2 and 10.3%, respectively) and suitable slope for PA (contributing 9 and 10.6%, respectively) stand out as key factors promoting activity intensity. In the walking model, the impact of non-rain/snow days (10.4%) remains significant, but the effects of elevation (4.5%) and suitable slope (6.2%) are less pronounced.

In terms of BE, the three models show relative consistency. The NDVI (with contributions of 9.5, 7.2, and 11.6%) and water body coverage area (with contributions of 7.3, 7.1, and 11.4%) are the most important factors promoting PA intensity. Population density, which served as a major limiting factor for PA willingness, no longer plays a significant role in limiting PA intensity.

At the SE level, economic development (whether group or individual) no longer shows a significant impact on PA intensity. However, consistent with the PA willingness model, education level remains the most important factor promoting PA intensity. Child-rearing also continues to be a significant limiting factor, ranking 6th in the overall model and 4th in the walking model.

## 4 Discussion

### 4.1 Comprehensive interpretation of the model results

In terms of variable contribution values and rankings, the running model and the overall model show a high degree of consistency, whereas the cycling and walking models differ significantly from the first two. This discrepancy becomes even more pronounced in the activity intensity models. On one hand, this reflects the fact that the driving factors for overall mobile PA differ from those for specific types of activity, and that the driving or limiting factors vary considerably between different types of activities. On the other hand, the characteristics of the dataset itself cannot be ignored. As seen in the activity frequency metrics in Table 2, the check-in data for running in Keep App far exceeds that for cycling and walking, which may explain the higher consistency between the running model and the overall model.

#### 4.1.1 Influence on willingness and intensity

Both overall and specific activity types show distinct differences in the factors influencing PA willingness and intensity. SE factors are the primary drivers of public participation in outdoor mobile PA, but their influence on activity intensity decreases significantly. Once individuals decide to engage in PA, GE and BE become the key factors determining activity intensity, which, to a large extent, determines the ultimate effectiveness of PA in improving public health.

Expanding further, in terms of PA willingness, the significant impact of economic development levels and education aligns with the findings from previous “small data paradigm” studies (5, 48). However, in terms of PA intensity, the impact of economic development levels is no longer evident, a finding that has not been addressed in prior research. Additionally, at the macro level, family structure factors, such as child-rearing, also demonstrate significant limiting effects. Improving essential public facilities and services aimed at families could be an important way to enhance public physical activity levels.

In terms of BE factors, NDVI and proximity to water bodies show a significant positive correlation with mobile PA, consistent with previous research findings (8, 32, 49). A notable difference is that accessibility (measured by road area density) does not show a significant impact on either willingness or intensity of PA at the macro scale. This might be due to the dataset reflecting more on regular physical activities and less on commuting-related activities. Furthermore, these macro-scale conclusions do not contradict micro-scale measures such as improving road connectivity, road density, or sidewalk density.

Previous studies have shown a complex effect of population density on residents’ physical activity (some studies indicate a positive effect, while others show the opposite) (18). According to the findings of this study, population density at the macro scale has a moderate negative impact on both PA willingness and intensity, which contrasts with micro-scale studies that suggest high-density neighborhoods promote walking (18). This could be because, at the micro scale, high-density neighborhoods typically have better infrastructure, which supports PA.

**Figure 7:**
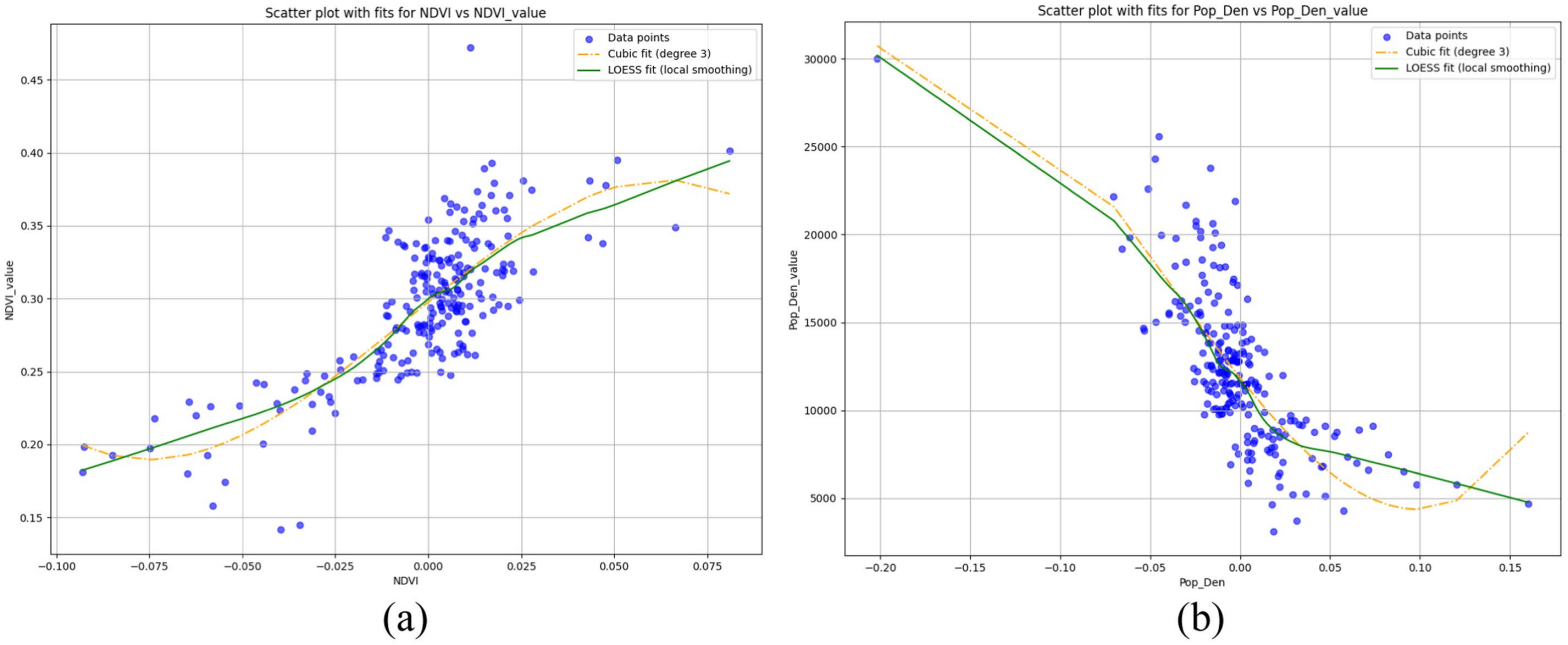
Nonlinear effects of variables in the overall model: **(A)** NDVI (NDVI_value represents the actual NDVI value of each sample; NDVI represents the shapley value of NDVI for each sample.); **(B)** Pop_Den (Pop_Den_value represents the actual population density value of each sample; Pop_Den represents the shapley value of population density for each sample).

It’s worth noting, as with previous research (9, 33, 50), that some indicators, such as NDVI and population density, exhibit clear threshold effects. Using the results of the overall model as an example, we apply Locally Estimated Scatterplot Smoothing (LOESS) and Cubic Polynomial Fit (Cubic fit) to fit the SHAP values and actual values of the samples in order to express this nonlinear effect (Figure 7). When the city’s NDVI index is below approximately 0.15, greening levels tend to suppress physical activity. When the index exceeds 0.35, greening levels consistently promote physical activity. However, within the 0.15–0.35 range, the effects are more complex, with both positive and negative SHAP values mixed. This suggests that low levels of greening may suppress PA to some extent, while the marginal effect of greening decreases as the index increases, indicating diminishing returns in promoting PA. The conclusions for population density align with those for greening. As a suppressive factor, high population density exhibits a clear inhibiting effect on PA. Once population density decreases to a certain threshold, this suppressive effect turns into a promotive one, although the rate of improvement gradually slows as density decreases further.

In terms of GE factors, for PA willingness, elevation (AL) and unsuitable wind force scale (UW) are two strong inhibitory factors, and their suppressive effects are consistent across different types of PA (51). When it comes to activity intensity, elevation (AL) continues to exhibit a significant negative influence, particularly in the overall model and the running model. Compared to PA willingness, the impact of terrain (unsuitable exercise slope) and precipitation (non-rain/snow days) increases substantially, indicating that lower precipitation and flat terrain have a significant positive effect on PA intensity (6, 7).

#### 4.1.2 Influence on different types of PA

Regarding the differences between various types of PA, child-rearing significantly suppresses overall PA willingness, but its effect is not pronounced in the specific activities of running, cycling, or walking. The reasons behind this may require further research. Meanwhile, the promotive effect of education levels strengthens progressively across these three types of activities. We speculate that this might be related to the “threshold” of each activity. Based on previous studies, higher education levels significantly increase public awareness of health and their subjective willingness to improve it (5, 48). However, when it comes to choosing an activity type, this willingness may be constrained by the activity’s intensity and the need for equipment or facilities. This pattern is also observed with the promotive factor of non-rain/snow days.

Interestingly, inhibitory factors such as elevation (AL), unsuitable wind speeds (UW), and child-rearing (CHILDREN) display opposite characteristics. This may be related to the preferences and motivations of individuals engaged in different types of activities. For example, due to the higher “threshold” of running, people with a regular running habit may have a relatively stable willingness to engage in PA, which can diminish the influence of inhibitory factors. In contrast, activities like walking, which have higher public participation, tend to exhibit the opposite effect, where these inhibitory factors have a more noticeable impact.

In conclusion, the interaction between PA type differences and intervention factors is significant. Low-threshold activities, such as walking, are more easily influenced by external environmental and socioeconomic conditions, amplifying the positive effects of promotive factors and the negative effects of inhibitory factors. In terms of PA willingness, this interaction is evident across all three categories: SE, BE, and GE factors. However, for PA intensity, this effect is only significant for SE and BE factors. The influence of GE factors remains relatively constant and is not amplified by the changing thresholds of different PA types.

#### 4.1.3 Comparison with conclusions from “micro-scale” and “small-sample” studies

**At the SE level**, small-sample studies have demonstrated the following:

1. **Barriers for lower socioeconomic groups**: Individuals with lower socioeconomic status are less likely to engage in free PA such as running and walking, due to pressures such as competition or child-rearing responsibilities (52, 53).
2. **Impact of education levels**: Lower levels of education may result in insufficient awareness of the health benefits of PA, thereby affecting willingness to participate (54).
3. **Facility access and inequality**: Research in developed countries has highlighted that lower socioeconomic groups often experience reduced PA levels due to a lack of facilities, making this a key focus of socioeconomic inequality (55). Additionally, findings from studies conducted at micro-spatial scales support these observations. High housing prices and high-profile communities have been shown to significantly promote physical activity, as wealthier residents not only enjoy better infrastructure but also have more leisure time for exercise (8, 9, 15).

These conclusions align with the findings of this study, which, through a large-sample analysis, identified the influence of economic development levels, education levels, and family structure factors (e.g., child-rearing) on PA willingness. However, it is worth noting that the diminished influence of socioeconomic factors on activity intensity, as revealed in this study, has not been explicitly addressed in prior research. This discrepancy may stem from existing studies not deliberately distinguishing between the concepts of activity willingness and activity intensity.

**At the GE level**, a review of small-sample studies on the impact of seasons and weather on PA reveals that weather conditions such as temperature, precipitation, and wind speed can serve as actual barriers to PA or as perceived barriers (e.g., subjective perceptions of being too cold or too hot) (7). These findings are consistent with this study’s conclusions regarding the directional impact of climate factors on PA.

However, there are two notable gaps in the existing literature:

1. **Relative importance of factors**: No studies have explicitly clarified the relative importance of these weather-related influences among various factors, especially when confounding factors are present. This ambiguity complicates the prioritization of resource allocation in policy-making.
2. **Limited research on elevation and slope**: Few studies have specifically explored the impact of factors such as elevation and slope on physical activity, leaving a gap in understanding the role of these geographical characteristics.

**At the BE level**, the following observations emerge from the comparison of micro-scale and macro-scale findings:

1. **Key factors at the micro scale**: Accessibility (road density, intersections, public transportation), design (greening, parks, water bodies), density (population and building density), and subjective perception (visual landscape variables based on street-view imagery) are the primary factors used to measure the impact of the built environment on PA (9).
2. **Macro-scale findings on NDVI and water environments**: At the macro scale, NDVI and proximity to water bodies have shown a significant positive association with mobile PA, aligning with most micro-scale studies (9, 17, 19). However, a study in Beijing demonstrated notable spatial heterogeneity in the attractiveness of water bodies, especially compared to water-rich southern Chinese cities (9). This suggests that while water environments are important for PA, in areas with limited water resources, people may seek other high-quality environments. This partially explains the conclusion of this study that water bodies have a promotive effect but are not a high-priority factor at the macro scale.
3. **Nonlinear effects of greening**: Consistent with micro-scale studies, greening exhibits significant nonlinear effects and thresholds in its influence on PA. Low-quality greening shows a clear inhibitory effect on PA (9, 19, 29), which will be discussed further later.
4. **Density’s effect on PA**: Micro-scale studies suggest that moderate population density promotes PA, while high density can lead to insufficient facilities and increased risk of injury, thereby suppressing PA (32, 56). Additionally, lighting and a sense of security are highly correlated with jogging preferences, especially in studies on older adults and children (30, 32, 57). These findings align with this study’s conclusion that high population density suppresses PA, but as density decreases, the suppression turns into promotion. However, further reductions in density result in diminishing positive effects, possibly due to residents’ perceived sense of safety.
5. **Inconsistent findings on accessibility**: At the macro scale, accessibility shows no significant effect on either PA willingness or intensity, differing from micro-scale findings. At the micro scale: (1) Streets are crucial for PA, whether through small-sample field studies or large-sample analyses (8, 58, 59); (2) Transportation accessibility (proximity to bus or subway stations) correlates strongly with cycling but not with running (8). This discrepancy may be related to the dataset used in this study, which primarily reflects leisure physical activities (regular activities with an exercise purpose), a hypothesis supported by the above findings.

#### 4.1.4 Explanations on the nonlinear and threshold effects

As previously mentioned, low-quality greening have a clear inhibitory effect on PA. We hypothesize that the complex influence observed in the NDVI range of 0.15–0.35 may be related to the quality of greening. Generally, as an essential component of urban infrastructure, the quality of greening is expected to correlate with the level of economic development in a city. Based on this assumption, we further analyzed the data generated by the model (using the Overall_willingness model as an example).

**Figure 8:**
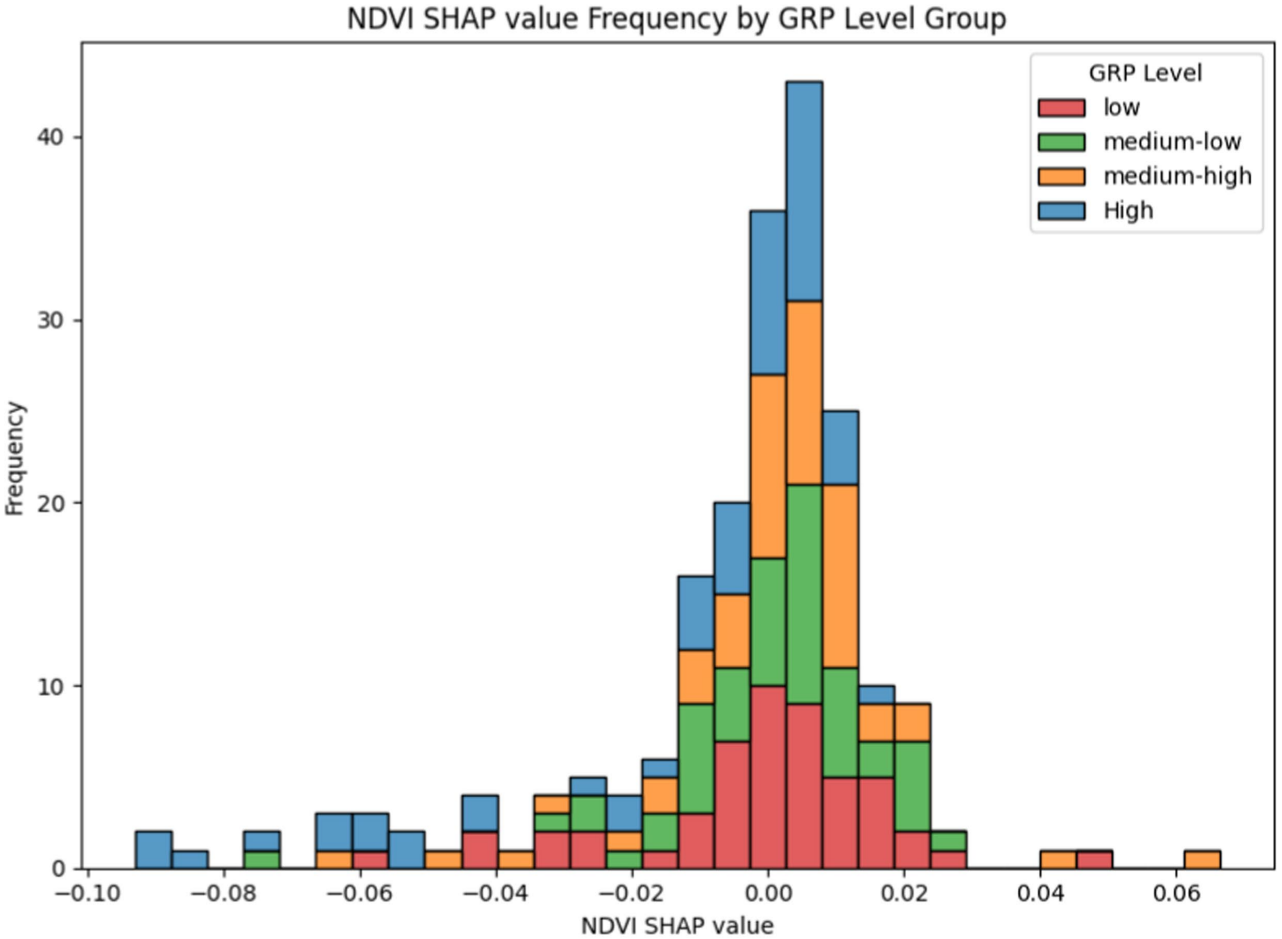
NDVI contribution distribution under the GRP perspective. The X-axis represents the Shapley values of NDVI for the samples, where the magnitude of the Shapley value indicates the contribution level of NDVI to PA for the sample, and the sign (positive or negative) indicates whether the NDVI level promotes or inhibits PA. The Y-axis represents the frequency of samples appearing within the corresponding X-axis intervals.

We sorted the samples based on their actual GRP values in ascending order and divided them into four groups (low, medium_low, medium_high, and high) using 25% of the sample size as the interval. A frequency distribution histogram was then created, with the Shapley values of the samples as the horizontal axis and the sample frequency as the vertical axis. The resulting sample frequency distribution histogram is shown in Figure 8:

From Figure 8, the following observations can be made:

1. **Low Economic Level Group**: The sample distribution is wide, with a notable proportion of negative values, indicating significant uncertainty in the contribution of greening to PA.
2. **Medium Economic Level Group**: The sample distribution is narrower, with peak values concentrated slightly on the positive side of zero, suggesting that in medium economic level regions, greening has a relatively balanced impact on PA.
3. **High Economic Level Group**: The distribution is noticeably skewed to the left, with a higher proportion of negative values, indicating that in high economic level regions, the promotive effect of greening on PA is somewhat suppressed.

We attribute the above results to differences in green space types and greening levels. Previous studies have shown that in China, economically developed areas typically have higher green space coverage and more diverse green space types, with significant differences in the number and area of parks being particularly notable (60). In less developed areas, due to economic and social constraints, green space coverage is lower and primarily dominated by residential and street greening, with limited connectivity (61). This partially explains the mitigating effect of greening initiatives on PA within groups characterized by high economic status. Studies examining the influence of newly constructed or enhanced parks (including amenities such as outdoor gyms, picnic areas, walking paths, playgrounds, irrigation systems, and landscaping) on PA have revealed that only 22% demonstrated a positive impact, 7% indicated no significant change, while some studies reported a decrease in both PA and park utilization following these improvements (62). Similarly, research conducted in Bogotá, Colombia, found that a higher ratio of park area was associated with reduced usage of mobile PA facilities, such as bike lanes (21).

Considering that parks in high economic level groups often include plazas, outdoor fitness equipment, and other recreational facilities, and given the cultural tendency in Asia toward collective physical activities (30), along with the relatively high population density in these regions, mobile PA may be absorbed into these spaces (e.g., individuals opting for stationary activities such as dancing or ball sports). This could explain the observed suppressive effects in certain samples. It is important to note that mobile PA represents only one form of PA, and its absorption does not negate the overall promotive role of park green spaces in encouraging PA.

**Figure 9:**
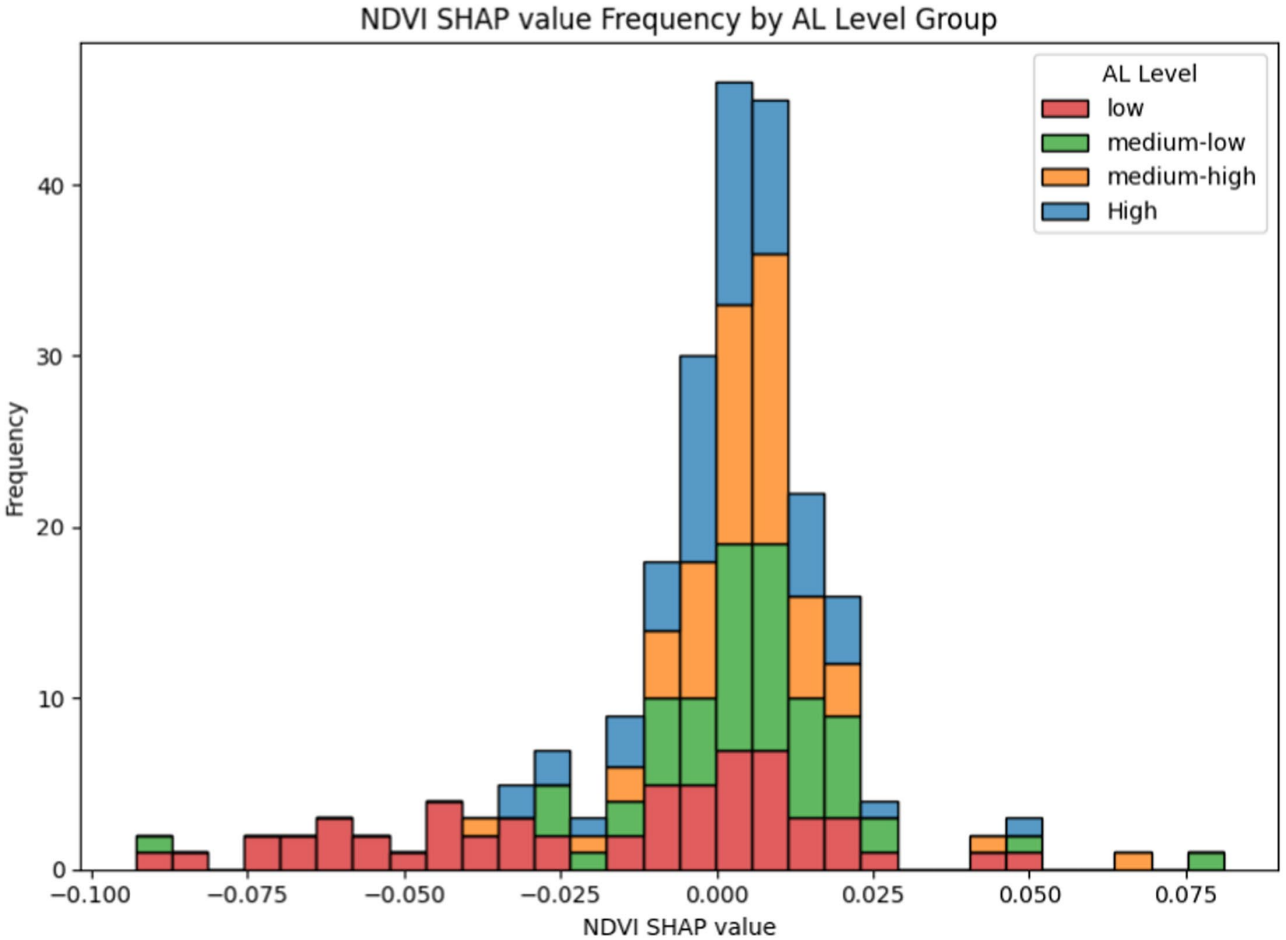
NDVI contribution distribution under the AL perspective. The X-axis represents the Shapley values of NDVI for the samples, where the magnitude of the Shapley value indicates the contribution level of NDVI to PA for the sample, and the sign (positive or negative) indicates whether the NDVI level promotes or inhibits PA. The Y-axis represents the frequency of samples appearing within the corresponding X-axis intervals.

Meanwhile, we further examined the interaction between NDVI and GE factors to verify the above conclusions and to ensure the effectiveness of greening optimization in regions with varying GE levels. Using the AL indicator as an example, we observed all samples, using the Shapley values of NDVI as the horizontal axis and the frequency of occurrence under each contribution level for the four altitude groups as the vertical axis. The resulting frequency histogram is shown in Figure 9. From the figure, we can conclude that for high-altitude and medium-altitude groups, the contribution of NDVI to PA is relatively stable and predominantly positive. This indicates that increasing appropriate greening in high-altitude regions has a positive effect on promoting PA. However, in low-altitude regions, the contribution of greening to PA varies significantly and is mostly negative, resembling the distribution observed in the high-economic-level group in Figure 8.

**Figure 10:**
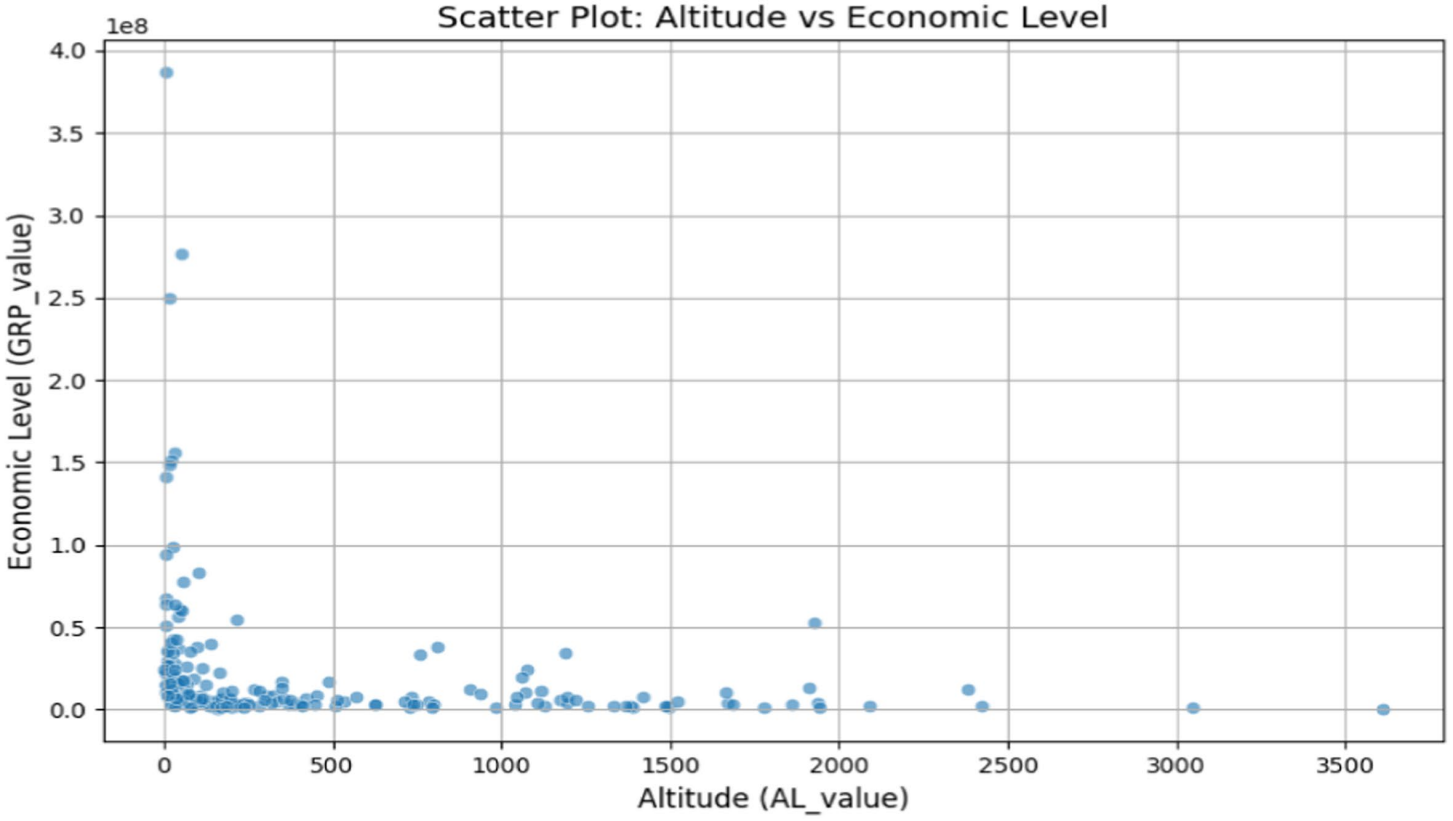
The distribution relationship between GRP and AL values.

To further investigate, we compared GRP and AL values across the entire sample and generated the distribution map shown in Figure 10. The figure reveals that high-altitude regions generally exhibit lower levels of economic development, indicating that the observations from the altitude perspective align with the conclusions drawn from the economic development perspective.

### 4.2 Policy recommendations and strategies

As mentioned earlier, intervention strategies from the perspective of PA are a key focus in the field of urban and rural planning. Given that previous research has primarily concentrated on BE perspectives at the level of individual cities or regions, planning strategies have typically been centered on two main aspects: 1. **Guideline Issues**: such as optimizing street networks, improving accessibility and public transportation convenience, and enhancing the quality of sports service facilities and the environment; 2. **Technical Indicator Issues**: such as identifying appropriate ranges for population density and building density indicators (18).

However, there are two key challenges: 1. The limitations of studies focused on individual cities may raise questions about the broader applicability of strategies, especially technical indicators; 2. From a cost-effectiveness standpoint, it remains unclear whether optimization strategies targeting BE factors are the most optimal intervention solution. Urban planning, as a tool for allocating public resources and shaping the physical environment, should adopt a broader perspective, aiming to maximize resource value within reasonable boundaries. Based on the findings of this study, which highlight the interaction between activity type differences and intervention factors, we propose the following policy recommendations and strategies. These are grounded in the principle of rational resource allocation and take into account the SE, GE, and GE environment.

#### 4.2.1 Prioritization in public resource allocation decisions

It is well-known that willingness to engage in PA is a prerequisite for achieving activity intensity, and socioeconomic factors are key to enhancing this willingness. This may suggest, especially for economically underdeveloped cities (or small towns), prioritizing public resource investment in economic development and social public services, rather than sports infrastructure, could be a more effective way to promote physical activity. **On one hand, the low level of urban governance caused by underdeveloped economies can have a significant negative impact on the public’s willingness to engage in PA, thereby limiting the effectiveness of policies aimed at promoting PA.** A review study on Latin American cities showed that while some initiatives in cities such as Mexico City, Rio de Janeiro, and Santiago de Chile seem to have had a positive impact on physical activity, widespread violence and insecurity in the region may influence physical activity patterns, particularly in impoverished areas (63). **On the other hand, given the “threshold differences” among different types of PA and their interactions with influencing factors, excessive investment in specialized sports facilities in these underdeveloped areas could lead to resource waste.** A quality evaluation study on public sports service facilities (mainly specialized sports venues) in small towns or counties in China showed that the variety and configuration of county-level public sports facilities already reveal issues of irrational allocation or resource overuse (64).

As China has long advocated for “focusing on economic development”, it is undeniable that economic growth plays an irreplaceable role in enhancing social governance, improving public services, and raising the quality of life. While the direct impact of economic development on PA remains a subject of discussion, its positive influence on overall societal progress is beyond question. Therefore, this paper does not emphasize the widely acknowledged importance of promoting economic development but instead focuses on the allocation of resources to enhance PA, offering the following recommendations for policymakers:

1. **Prioritize measures that integrate well with public service facilities.** For example, improving urban greening levels and quality, increasing public park facilities, expanding slow traffic systems (such as bicycle lanes), and enhancing urban safety (21, 63). While this paper does not specifically discuss the impact of public transportation on physical activity, experiences from Latin America and China suggest that improving the accessibility of public transportation systems (e.g., Bus Rapid Transit [BRT], subways) and reducing dependence on motorized transport (especially private cars) due to urban sprawl are effective measures to promote public physical activity levels (21, 65, 66).
2. **Focus on health equity compensation for low-income groups.** Urban policies should allocate public health resources preferentially to vulnerable groups, such as low-income individuals, the older adult, and children (67, 68). The findings of this study, particularly the “inhibitory effect of low-quality green space on PA”, further support this point. Urban renewal projects in cities like Guangzhou, Shanghai, and Chongqing, such as the “Old Community Renewal Program” and “Urban Village Transformation”, have improved public health resource accessibility for disadvantaged residents through updates to public service facilities, green spaces, and infrastructure (69). A case study from South Africa also corroborates this perspective (70).
3. **Prioritize investment in “low-barrier, high-participation” facilities.** When selecting public sports facilities, resources should focus on facilities for walking and light PA (e.g., walking paths, jogging tracks, and low-intensity outdoor exercise facilities). Such facilities have low participation barriers, amplifying the positive effects of promotive factors on PA. Additionally, they can integrate easily with streets and public transportation systems, positively influencing commuting-related PA. Experiences from Guangzhou, Shenzhen, and Chengdu in China indicate that residents living near greenways show higher enthusiasm for PA (69). Similarly, the Paris Olympics urban greenway and large-scale natural experiments in the UK demonstrate that proximity to new walking and cycling routes is associated with increased PA (62). This has also been validated by experiences in Latin America (71, 72).

Finally, considering that the “contradiction between economic development and social justice” is a perennial theme in urban studies (73), we recognize that Health Impact Assessment (HIA) could offer valuable insights for better balancing investment between economic development and PA promotion, particularly in economically underdeveloped areas. Empirical studies on the implementation effects of HIA across more than 30 European countries and regions with diverse economic, administrative, and social contexts have revealed that HIA, whether applied at the urban level (e.g., healthy city planning and transportation planning) or the neighborhood level (e.g., urban design and renewal), addresses cost-effectiveness and sustainability in two key ways. First, it helps alleviate the economic pressure of health policies by balancing expenditures and benefits. Second, it facilitates equitable compensation for vulnerable groups in urban development (71, 72).

#### 4.2.2 Strengthen the enhancement of school physical education and community public services

Based on the significant impact of SE factors such as child-rearing and education level on both PA willingness and intensity, it is essential to strengthen relevant social public services to enhance public PA levels. Since “universally improving national education levels” is already a global consensus, it does not require further emphasis here. Instead, we propose increasing the importance of physical education in schools and strengthening PA promotion and education at the community level as further recommendations to boost public PA. On the one hand, several countries, including the United States, the United Kingdom, and Ireland, have successfully improved students’ PA participation through school-wide PA promotion programs (74, 75). Moreover, studies have shown that individuals who participate in organized school sports are three times more likely to remain regularly active in adulthood compared to others (76).

On the other hand, community support has been proven to be an effective means of promoting PA (4, 5). Community support activities, including targeted education and outreach, using various media channels for promotion, and providing health advisory services for community members, have shown positive impacts in places such as Hangzhou, Japan, and the United States (77).

In summary, considering the diversity of the selected sample in this study (with significant differences in economic development levels and natural geographical conditions across cities) and the consistency of the experimental conclusions with similar studies worldwide, we believe that the above recommendations align well with global public health strategies. It is worth noting that, given the significant impact of economic, social, and cultural differences on individuals’ willingness to participate in PA (5), local governments should further strengthen field investigations and public participation when formulating detailed and actionable policies. This approach ensures that measures are tailored to specific local conditions and adapted to the unique characteristics of each city.

### 4.3 Outlook and limitations of this study

#### 4.3.1 Limitations of this study

This study has several limitations that require further exploration. First, the user group in this study is concentrated in the 15–40 age range, which limits the applicability of the conclusions to a broader demographic (such as including the older adult and adolescents). However, existing studies have shown that the inhibitory effect of high population density on physical activity and the promotive effect of greening levels are consistently applicable to both older adult and adolescent groups (58, 78, 79). Additionally, given that adolescent PA is often school-organized and older adult individuals have a heightened need for a sense of safety (30, 80), policy recommendations should be tailored to meet the specific needs of these groups. Future research should incorporate more comprehensive sample data to further validate the applicability of these conclusions. Additionally, the lack of access to personal data (such as age, income, and gender) restricts the analysis of individual-level preferences and motivations.

Secondly, due to the lack of time-series data, the study is unable to assess the impact of time periods on PA. However, this study uses indicators of suitable climate conditions (such as suitable temperature days, rain/snow days, etc.) to replace annual averages, aiming to minimize the impact of seasonal differences on the study results. Existing literature suggests that this design can partially compensate for the lack of time-series data, especially regarding the influence of climate factors on PA (7). However, diurnal factors such as day length may have additional effects on jogging behavior and perceived safety, which can only be further explored in future research. Moreover, time periods (such as differences between weekdays and weekends) and mobility patterns (such as one-way versus round-trip) may influence the ranking of variable importance, but they do not change the positive or negative contributions of the variables (11). Therefore, since this study primarily focuses on leisure physical activities rather than periodic commuting activities, the impact of temporal factors on the conclusions is relatively minor.

Finally, due to the lack of shared bicycle data, this study is unable to effectively explain the environmental preferences for cycling behavior. Existing studies have shown that cycling behavior significantly differs from other types of physical activity in terms of its nature. Cycling behavior is more often associated with commuting, particularly on weekdays, where office-related Points of Interest (POIs) have the most significant contribution to cycling behavior, while leisure-related POIs are more influential during non-working days (29, 46). Commuting cycling requires high road density, road classification, and good integration with public transportation (81, 82), whereas the dataset used in this study mainly reflects leisure physical activities (such as daily exercise), which differs significantly from commuting behavior. Therefore, the results of this study are more applicable to explaining mobile PA aimed at leisure and exercise rather than commuting behaviors. Future research could further refine the analysis of the impact mechanisms of commuting and recreational cycling by incorporating shared bicycle data or conducting field surveys.

#### 4.3.2 Potential future research directions

(1) **Expand Data Sources:** Future research could integrate data from other fitness apps or smart devices to expand the sample size, covering a wider age range and populations from different cities and regions to improve the generalizability of the findings. Additionally, incorporating time-series data could allow for the exploration of long-term impacts of seasonal changes and extreme weather on physical activity.
(2) **Conduct Subgroup Analyses:** This study encompasses a broad sample range, from international metropolises to economically underdeveloped areas, and from flatland cities to mountainous ones. While this complex sample set aids in identifying general trends and phenomena, it poses challenges in forming specific, targeted planning strategies. Future studies could conduct further classification analyses of the sample cities, focusing on economically developed regions, underdeveloped areas, flatland cities, mountainous cities, etc.
(3) **Explore Differences in Micro and Macro-Scale Findings:** Given the differences between the findings of this study and micro-scale results, further investigation is needed to understand the underlying reasons for these discrepancies and to better address the integration of macro and micro-scale factors in policy-making.

## 5 Results

This study conducted a machine learning regression analysis on regular mobile physical activity data from 290 cities in China, examining the impact of GE, SE and BE factors on public PA willingness and intensity. The findings reveal differences between various types of PA (such as running, cycling, and walking) and their influencing mechanisms:

First, PA willingness is primarily driven by SE factors. Higher levels of economic development and education significantly increase public participation in PA. PA intensity, on the other hand, relies more on natural geographic conditions and the built environment. Favorable climatic conditions (such as low elevation, suitable temperatures, and wind speeds) and urban greening significantly enhance activity intensity.

Second, the study reveals the interactive relationship between PA types and influencing factors. Low-threshold activities (such as walking) are more affected by external environmental and socioeconomic conditions, amplifying the positive effects of promotive factors and the negative effects of inhibitory factors. In terms of PA willingness, all three factor categories—SE, BE, and GE—exhibit this interactive relationship. However, in terms of PA intensity, this effect is only significant for SE and BE factors, with GE factors remaining relatively constant and unaffected by activity thresholds.

Third, at the macro scale, certain influencing factors exhibit significant nonlinearity and threshold effects. For example, when the NDVI level is below 0.15, low levels of greening have an inhibitory effect on physical activity. When it exceeds 0.35, it shows a promotive effect, and this promotive effect exhibits obvious marginal effects.

Finally, by comprehensively analyzing the factors influencing both PA willingness and intensity, the study offers planning and policy recommendations from the perspective of public resource allocation.

## Data Availability

The raw data supporting the conclusions of this article will be made available by the authors, without undue reservation.

## Footnotes

^1^Download Website: www.keep.com.

^2^ www.openstreetmap.org

^3^ www.gscloud.cn

^4^ waptianqi.2345.com

^5^ www.macrodatas.cn
